# P-372. Accelerated Hyperglycemia in Persons with HIV Started on Dolutegravir, A hypothesis Building Insulin Kinetic Study

**DOI:** 10.1093/ofid/ofaf695.590

**Published:** 2026-01-11

**Authors:** Frank Mulindwa, Jean-Marc Schwarz

**Affiliations:** United Health Services, Wilson Hospital, Johnson City, NY; University of California San Francisco, San Francisco, California

## Abstract

**Background:**

Over 80% persons living with HIV (PLHIV) in low and middle-income countries were taking fixed dose combination tenofovir disoproxil fumarate/lamivudine/dolutegravir (TLD) by mid-2024. Despite a very good side effect profile of dolutegravir (DTG), there have been multiple case reports of PLHIV developing hyperglycemia within weeks to a few months after switching to DTG.

**Table 1.**
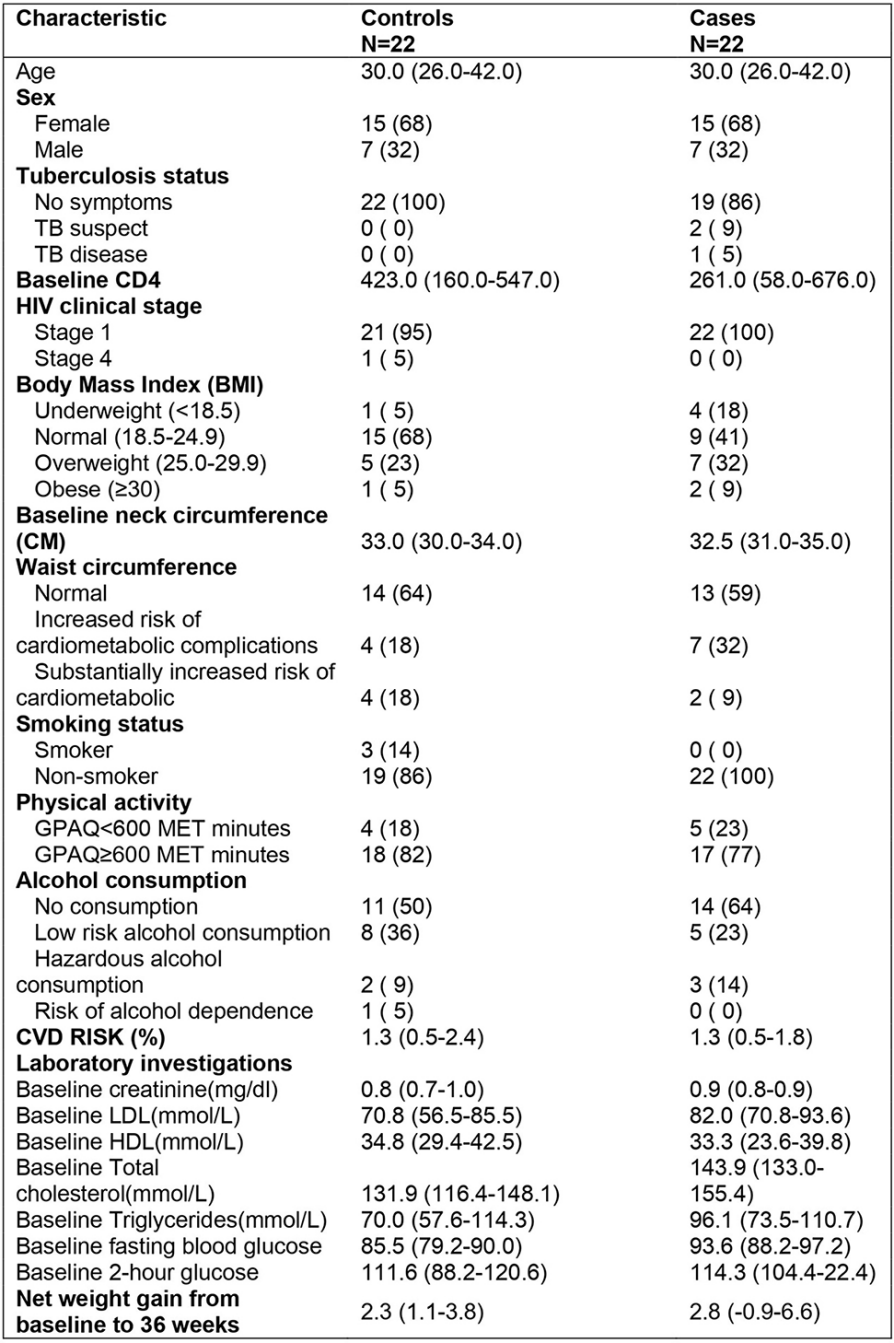
Baseline clinical and demographic characteristics of participants with incident pre-diabetes mellitus at 36 weeks compared to those with normal blood glucose levels.

**Table 2.**
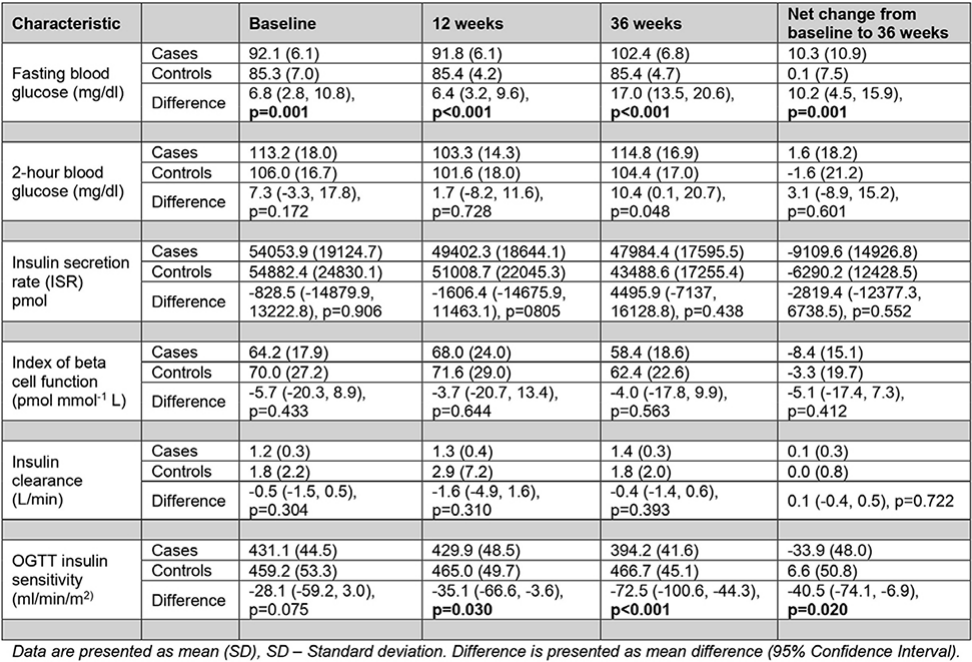
Comparison of changes in blood glucose and insulin kinetic parameters from baseline to 36 weeks between participants with incident pre-diabetes (cases) and those with normal glucose levels (controls)

**Methods:**

We analyzed data from the ‘Glucose metabolism changes in Ugandan PLHIV on Dolutegravir (GLUMED)’ study which was a prospective cohort study with ART naïve persons with HIV ≥ 18 years followed up on TLD over 48 weeks with oral glucose tolerance tests (OGTTs) performed at baseline, 12 weeks and 36 weeks. We measured serum glucose, insulin and C-peptide at 0,30,60,90 and 120 minutes of the OGTTs. OGTT insulin sensitivity and index of beta cell function were calculated from OGTT blood glucose, C-peptide and insulin. Insulin secretion rates (ISR) were calculated by deconvolution and insulin clearance rates were determined by dividing the ISR- area under curve (AUC) by the product of insulin volume of distribution. We compared glucose changes as well as the above insulin kinetic parameters over 36 weeks in 22 cases (PLHIV who developed incident pre-diabetes at 36 weeks) and 22 controls (PLHIV who had normal glucose over 36 weeks).

**Table 3.**
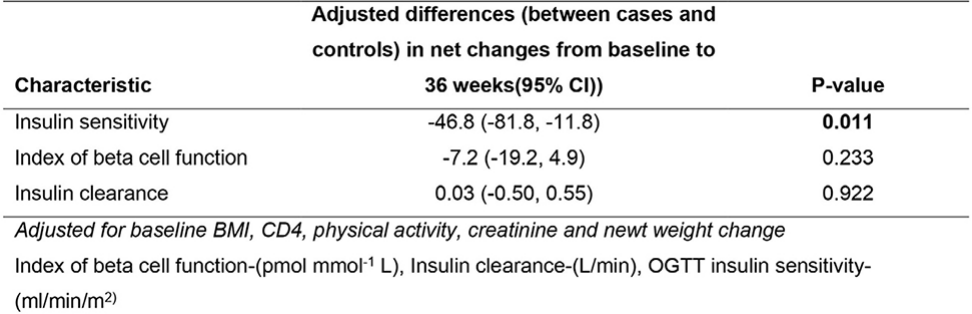
Adjusted differences in changes over 36 weeks in oral glucose tolerance insulin sensitivity, index of beta cell function and insulin clearance compared between cases and controls

**Figure 1:**
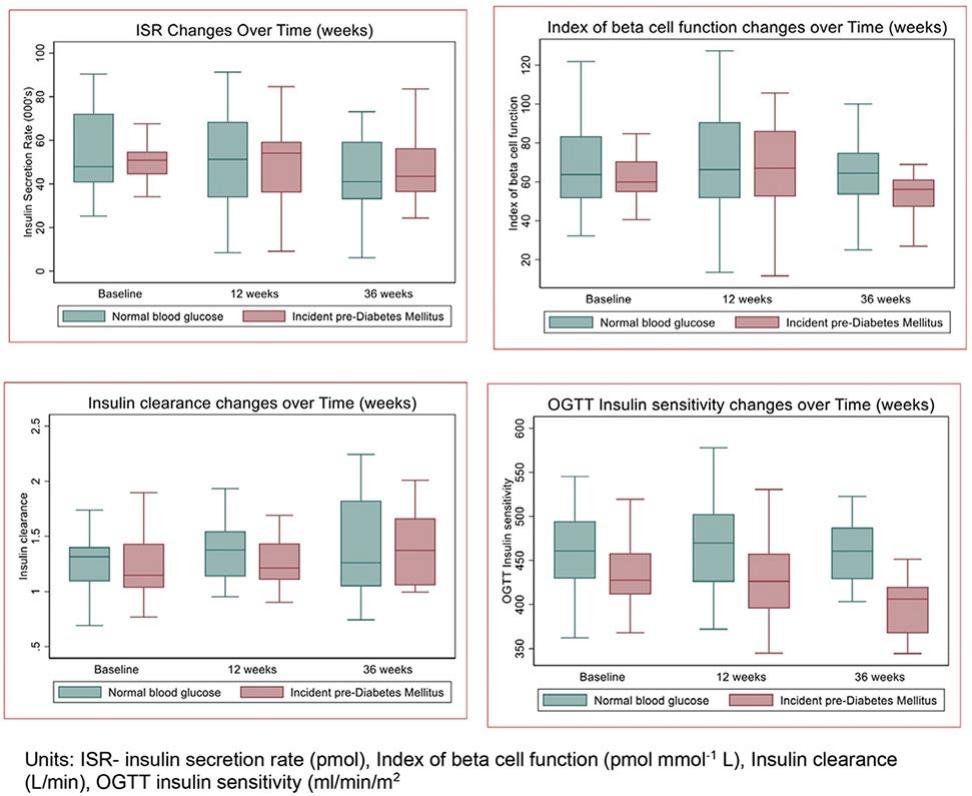
Changes in oral glucose tolerance test insulin resistance, beta cell function, insulin sensitivity and insulin clearance compared between cases and controls over 36 weeks

**Results:**

The mean age of participants was 30 years in both groups with 15 participants being female in both groups. We found more pronounced reduction in insulin sensitivity in the cases as compared to controls at 36 weeks (adjusted difference in changes in mean insulin sensitivity at 36 weeks from baseline between cases and controls (ml/min/m^2)^), 95%CI): -46.8 (-81.8, -11.8), p-value: 0.011) but no differences in changes in the index of beta cell function and insulin clearance.

**Conclusion:**

We hypothesize that the acerated hyperglycemia that is observed in a section of persons living with HIV when started on dolutegravir based anti-retroviral therapy is driven by impairment in insulin sensitivity. These patients may have an underlying yet to be determined, predisposition to impairment in insulin sensitivity that is accentuated on exposure to dolutegravir. More mechanistic studies may be warranted in ART exposed populations.

**Disclosures:**

All Authors: No reported disclosures

